# Decreased empathy response to other people’s pain in bipolar disorder: evidence from an event-related potential study

**DOI:** 10.1038/srep39903

**Published:** 2017-01-06

**Authors:** Jingyue Yang, Xinglong Hu, Xiaosi Li, Lei Zhang, Yi Dong, Xiang Li, Chunyan Zhu, Wen Xie, Jingjing Mu, Su Yuan, Jie Chen, Fangfang Chen, Fengqiong Yu, Kai Wang

**Affiliations:** 1 Laboratory of Cognitive Neuropsychology, Department of Medical Psychology, Anhui Medical University, Hefei, Anhui, China; 2 Collaborative Innovation Centre of Neuropsychiatric Disorders and Mental Health, Hefei, Anhui, China; 3 Anhui Mental Health Centre, Hefei, Anhui, China; 4 Anhui Province Key Laboratory of Cognition and Neuropsychiatric Disorders, Hefei, Anhui, China; 5 Department of Psychology, Southwest University, Chongqing, China; 6 Department of Neurology, The First Affiliated Hospital of Anhui Medical University, Hefei, Anhui, China

## Abstract

Bipolar disorder (BD) patients often demonstrate poor socialization that may stem from a lower capacity for empathy. We examined the associated neurophysiological abnormalities by comparing event-related potentials (ERP) between 30 BD patients in different states and 23 healthy controls (HCs, matched for age, sex, and education) during a pain empathy task. Subjects were presented pictures depicting pain or neutral images and asked to judge whether the person shown felt pain (pain task) and to identify the affected side (laterality task) during ERP recording. Amplitude of pain-empathy related P3 (450–550 ms) of patients versus HCs was reduced in painful but not neutral conditions in occipital areas [(mean (95% confidence interval), BD vs. HCs: 4.260 (2.927, 5.594) vs. 6.396 (4.868, 7.924)] only in pain task. Similarly, P3 (550–650 ms) was reduced in central areas [4.305 (3.029, 5.581) vs. 6.611 (5.149, 8.073)]. Current source density in anterior cingulate cortex differed between pain-depicting and neutral conditions in HCs but not patients. Manic severity was negatively correlated with P3 difference waves (pain – neutral) in frontal and central areas (Pearson *r* = −0.497, *P* = 0.005; *r* = −0.377, *P* = 0.040). Electrophysiological correlates of empathy processing are reduced in BD depending on manic symptom severity.

Bipolar disorder (BD) is a severe mood disorder characterized by alternating manic or hypomanic and depressive episodes. Manic or hypomanic episodes are characterized by elevated mood, irritable mood, or both, which are variable in severity and length, while depressive episodes are states of low mood^1^,^2^. Even in the asymptomatic phase (remission state), BD patients still exhibit functional disturbances, such as social cognition deficits^3^. Diagnosis of BD is always difficult, and BD is often misdiagnosed as unipolar depression^1^. The limitations of BD diagnosis have hampered research into possible cures and functional rehabilitation strategies.

Bipolar disorder is one of the major causes of disability worldwide and afflicts not only individual patients but also family and society by placing a heavy burden on mental health and other social services^4^,^5^. Bipolar disorder patients show disabilities in social and occupational functioning. Compared to healthy controls (HCs), BD patients have fewer social interactions^6^,^7^. Patients with BD also showed impairments in emotion identification regardless of current disease severity, a deficit also present in first-degree relatives, indicating a potential endophenotype for BD^3^,^8^,^9^. In addition, BD patients with varied disease severity demonstrated deficits in mentalizing^10^,^11^. These observations illustrate that BD patients are unable to comprehend and ascribe mental states of others, which could underlie deficits in empathy.

Empathy is defined as the ability to imagine oneself in another’s situation and to share their mental state^12^,^13^. The ability to empathize is crucial for social interactions^14^ and is closely related to emotion identification and mentalizing^15^. Although impaired empathic ability may be an important factor for social dysfunction in BD, research on empathy processing in BD remains scarce. However, the few studies addressing this issue indicated that empathy is impaired in BD patients. Compared to HCs, BD patients showed lower empathy scores on the Temperament and Character Inventory^16^; as well as lower scores in perspective taking and higher scores in personal distress on the Interpersonal Reactivity Index (IRI), a widely used test for empathy assessment^17^,^18^. Moreover, both BD patients and their first-degree relatives performed worse than HCs in a task assessing empathy^19^,^20^.

The neural mechanisms of empathy have also been explored in large scale studies, most focused on pain empathy due to the robustness of pain in inducing empathy. Functional magnetic resonance studies have reported that watching others in pain consistently activates regions of the anterior insula and anterior cingulate cortex (ACC)^13^,^21^. Moreover, neural processing of pain empathy can be modulated by engagement in a cognitive task^22^. Event-related potentials (ERP) are useful for examining the time course of pain empathy processing with millisecond precision. The neural mechanisms of pain empathy can be divided into two temporal phases, an early emotional phase automatically activated through perception of others in pain and a late cognitive phase that can be modulated on a conscious level^23^,^24^. Based on previous ERP studies on pain empathy, perception of pain in others is associated with distinct components, N1 with the early emotional sharing and P3 with later cognitive evaluation^23^,^24^.

To the best of our knowledge, there have been no studies investigating the neural activation patterns underlying pain empathy in BD. In previous studies, BD patients showed dysfunction in emotional processing and emotional regulation that were associated with abnormalities in the fronto-limbic neuroanatomical network^25^. Several studies found functional disruption in the amygdala, insular cortex, and ACC of BD patients while viewing emotional (especially negative emotional) pictures^26^,^27^. Furthermore, a meta-analysis found reduced grey matter in ACC and bilateral insula of BD patients^28^. These brain regions exhibiting functional disruption in BD patients also make important contributions to processing of pain empathy in healthy subjects.

The ERP components N1 and P3 are always induced by pain empathy tasks. Previous studies of BD showed abnormalities in N1, indicating a deficit in the early stage of sensory processing^29^. Another study showed increased N1 related to the attentional bias associated with reward-related risk-taking^30^. Decreased P3 amplitude has also been widely observed in BD patients. For instance, reduced P3 amplitude has been reported in BD patients during cognitive processing of an oddball task, self-referential processing, and emotion identification processing^29^,^31^,^32^. However, investigations of the neural processing abnormalities underlying pain empathy deficits in BD as revealed by ERP are still lacking.

The aim of the present study is to compare the neural processing of pain empathy between BD patients and HCs as manifested by ERPs. Both BD patients and HCs were asked to complete a pain empathy task involving images of individuals in pain and neutral conditions during ERP recording. Based on previous studies, we expected reduced N1 and P3 ERP amplitudes induced by the pain depictions relative to neutral images in BD patients compared to HCs. We also expected decreased activation in pain empathy-related brain areas, such as anterior insula and ACC, according to electrophysiological source analysis when perceiving others in painful situations. In order to identify whether a potential deficit of pain empathy is trait-related or state-related, we also investigated correlations with current symptom severity.

## Results

### Demographic information and assessment results

There were no differences in age, sex ratio, and years of education between BD patients and HCs. Both groups performed similarly in Verbal Fluency, Digit Span Forward, Digit Span Backward, and Stroop tests, but patients performed worse on the Montreal Cognitive Assessment. In the Chinese IRI, patients and HCs performed similarly on fantasy and personal distress items, while patients scored lower on perspective taking and empathy concern (Table 1).

### Picture assessments and behavioural results

Pain rating scores for pain depictions and neutral pictures differed significantly in an independent cohort of healthy subjects (*t*_32_ = 30.21, *P* < 0.01). There was a significant stimulus (neutral vs. pain) × task (pain vs. laterality) interaction for accuracy (*F*_1, 51_ = 11.634, *P* = 0.001). Pairwise comparison showed higher task accuracy for neutral images compared to pain depictions in both tasks [pain task (*F*_1, 51_ = 21.024, *P* < 0.001, *η*^2^ = 0.292); laterality task (*F*_1, 51_ = 19.274, *P* < 0.001, *η*^2^ = 0.274)] (Table 2).

Furthermore, there was a significant main effect of group (HCs vs. BD) in reaction time for correct answers (RTs) (*F*_1, 51_ = 18.513, *P* < 0.001, *η*^2^ = 0.266), with patients responding slower than HCs in all conditions. The interaction of stimulus × task also reached significance (*F*_1, 51_ = 4.485, *P* = 0.039, *η*^2^ = 0.081). Pairwise comparison showed that RTs were longer for neutral stimuli compared to pain depictions for the laterality task (*F*_1, 51_ = 62.400, *P* < 0.001, *η*^2^ = 0.550) but not for the pain task (Table 2).

There were no group differences in discrimination of pain ratings between painful depictions and neutral stimuli (Table 1).

### Electrophysiological results

**Figure 1:**
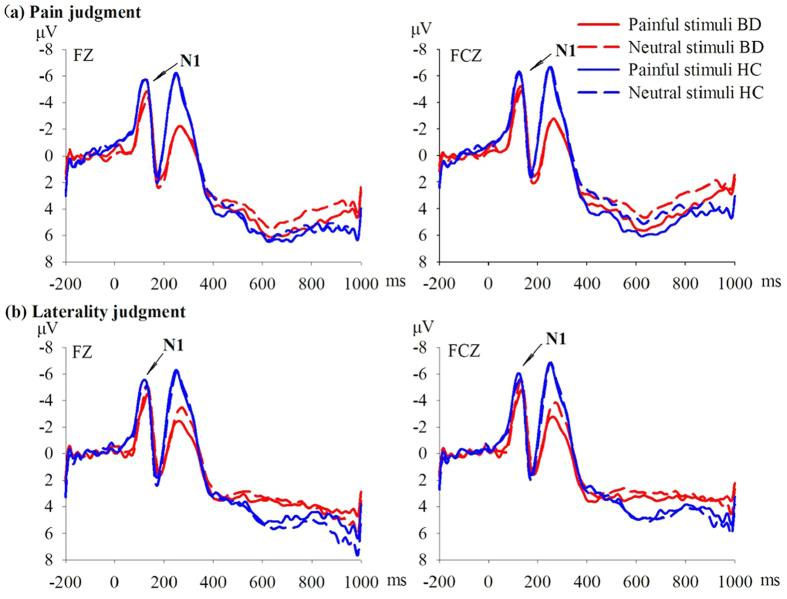
Grand average stimulus-locked waveforms for pain-depicting (solid lines) and neutral (dashed lines) images for both the healthy control (HC) group (blue lines) and the bipolar disorder (BD) group (red lines). Early ERP components at electrode FZ and FCZ are depicted. Stimuli onset is at time = 0 msec.

For the ERP component N1, repeated measures analysis of covariance (RM-ANCOVA) showed no main effect of stimulus. The interaction of stimulus, task, and group (BD vs. HCs) reached significance for the frontal area (*F*_1, 50_ = 7.366, *P* = 0.009, *η*^2^ = 0.128). Pairwise comparison showed that the difference between N1 amplitudes induced by pain depictions and neutral images reached significance (*F*_1, 50_ = 5.130, *P* = 0.028, *η*^2^ = 0.093), with BD patients showing less positive amplitudes during pain depictions than during neutral images. The difference was not significant in the HCs (*P* = 0.181) (Fig. 1).

**Figure 2:**
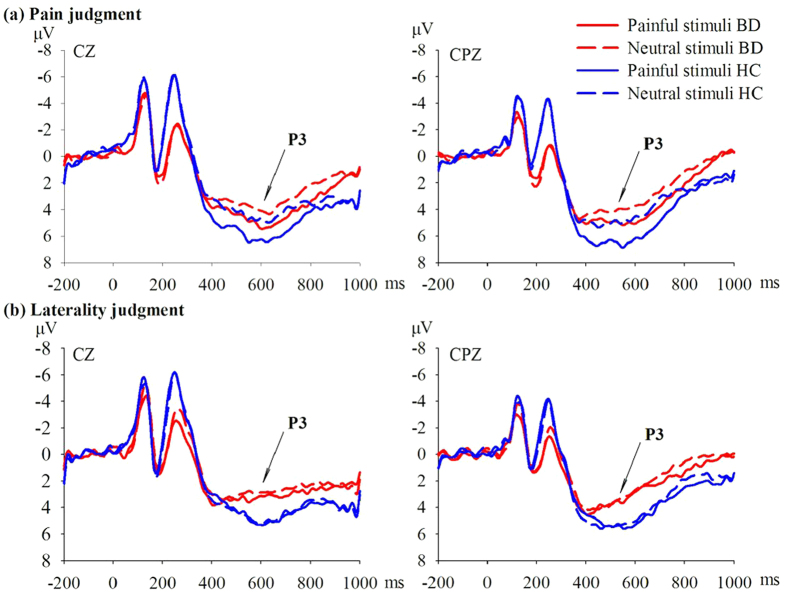
Grand average stimulus-locked waveforms for pain-depicting (solid lines) and neutral (dashed lines) images for both the healthy control (HC) group (blue lines) and the bipolar disorder (BD) group (red lines). Late ERP components at electrodes CZ and CPZ are depicted. Stimuli onset is at time = 0 msec.

For the early phase of P3 (450–550 ms), RM-ANCOVA revealed main effects of stimulus as measured from the central area (*F*_1, 50_ = 5.042, *P* = 0.029, *η*^2^ = 0.092). Amplitudes of P3 (450–550 ms) evoked by pain depictions were larger than those evoked by neutral images. Furthermore, the interaction of stimulus, task, and group reached significance for the frontal area (*F*_1, 50_ = 5.001, *P* = 0.030, *η*^2^ = 0.091) and central area (*F*_1, 50_ = 4.537, *P* = 0.038, *η*^2^ = 0.083), and marginal significance for the occipital area (*F*_1, 50_ = 3.515, *P* = 0.067, *η*^2^ = 0.066). Pairwise comparisons showed that pain depictions evoked P3 (450–550 ms) responses of greater positive amplitude compared to neutral images in the HCs group on the pain task for the frontal (*F*_1, 50_ = 8.772, *P* = 0.005, *η*^2^ = 0.149), central (*F*_1, 50_ = 12.824, *P* = 0.001, *η*^2^ = 0.204), and occipital areas (*F*_1, 50_ = 9.896, *P* = 0.003, *η*^2^ = 0.165), while positive P3 (450–550 ms) amplitudes did not differ significantly between stimuli for BD patients in any area (*P*_min_ = 0.284). Moreover, the group difference reached marginal significance for the pain depiction condition in the central area (*F*_1, 50_ = 3.824, *P* = 0.056, *η*^2^ = 0.071) and significance for the occipital area (*F*_1, 50_ = 4.371, *P* = 0.042, *η*^2^ = 0.080), but not in the neutral condition for any area (*P*_min_ = 0.319), with larger amplitudes in HCs than BD patients (Fig. 2).

For the later phase P3 (550–650 ms), RM-ANCOVA revealed main effects of stimulus in the frontal (*F*_1, 50_ = 4.362, *P* = 0.042, *η*^2^ = 0.080) and central area (*F*_1, 50_ = 5.192, *P* = 0.027, *η*^2^ = 0.094) and a main effect of group in the occipital area (*F*_1, 50_ = 4.910, *P* = 0.031, *η*^2^ = 0.089). Amplitudes in response to pain depictions were larger than in response to neutral images, and pain-associated P3 (550–650 ms) amplitudes were larger in HCs than BD patients. Furthermore, the interaction of stimulus, task, and group reached significance in the frontal area (*F*_1, 50_ = 4.235, *P* = 0.045, *η*^2^ = 0.078) and marginal significance in the central area (*F*_1, 50_ = 3.830, *P* = 0.056, *η*^2^ = 0.071)]. Pairwise comparisons showed that painful depictions evoked P3 (550–650 ms) responses of greater positive amplitude compared to neutral images during the pain task in HCs for the frontal area (*F*_1, 50_ = 13.262, *P* = 0.001, *η*^2^ = 0.210) and central area (*F*_1, 50_ = 19.383, *P* < 0.001, *η*^2^ = 0.279), but not in BD patients (*P*_min_ = 0.172). Moreover, the group difference reached significance in the pain depiction condition for the central area (*F*_1, 50_ = 5.562, *P* = 0.022, *η*^2^ = 0.100) but in no brain region on the neutral image condition (*P*_min_ = 0.520), with larger amplitudes in HCs than BD patients (Fig. 2).

### Source-localization data

**Figure 3:**
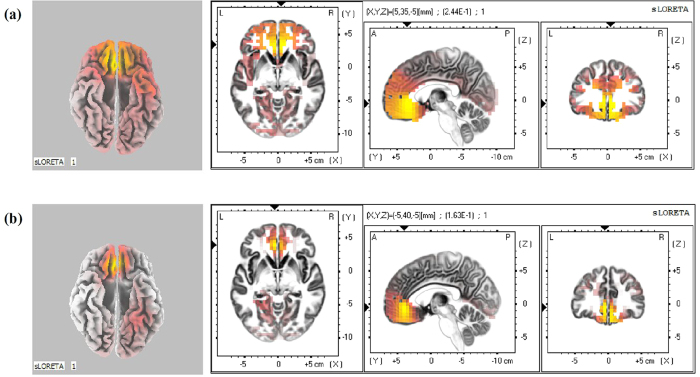
Current source density shows differences in the ACC upon presentation of between pain-depicting and neutral conditions around 550 ms and 650 ms in both groups ((**a**) for healthy control group and (**b**) for bipolar disorder group).

To compare the cortical regions involved in pain empathy processing between BD patients and HCs, voxel-based whole-brain standard low-resolution electromagnetic tomography images (sLORETA) were analysed in pain depiction and neutral image conditions during the later P3 phase (550 to 650 ms post-stimulus presentation) using non-parametric randomisation tests. As hypothesized, activation of brain regions involved in pain judgment was higher in HCs for the pain depiction condition than the neutral image condition (*t*_22_ = 0.182, *P* = 0.023), while the activation pattern was similar in both conditions for BD patients (*t*_29_ = 0.023, *P* = 0.266). During pain judgment, current source density in the ACC (Brodmann 32, max values obtained at x = 5, y = 35 and z = −5) was larger in the pain depiction condition than in the neutral image condition for HCs (Fig. 3).

### Analyses of medication effects

To investigate effects of medication on ERP results, we conducted RM-ANCOVA to compare the differences among medicated patients, un-medicated patients, and HCs. All four types of medication received by patients (antipsychotics, antidepressants, benzodiazepines, and mood stabilizers) showed interactions with stimulus condition and task for N1 measured from the frontal area [antipsychotics (*F*_2, 49_ = 3.706, *P* = 0.032, *η*^2^ = 0.131); antidepressants (*F*_2, 49_ = 3.667, *P* = 0.033, *η*^2^ = 0.142); benzodiazepines (*F*_2, 49_ = 3.915, *P* = 0.026, *η*^2^ = 0.138); mood stabilizers (*F*_2, 49_ = 3.981, *P* = 0.025, *η*^2^ = 0.140)]. Pairwise comparison showed that pain depictions in the pain task evoked less positive N1 amplitudes compared to neutral images in patients receiving antidepressants (*F*_1, 49_ = 4.550, *P* = 0.038, *η*^2^ = 0.085), benzodiazepines (*F*_1, 49_ = 5.900, *P* = 0.019, *η*^2^ = 0.107), and mood stabilizers (*F*_1, 49_ = 4.914, *P* = 0.031, *η*^2^ = 0.091). Response amplitudes induced by pain depictions were less positive compared to neutral images in the pain task for patients not taking antipsychotics (*F*_1, 49_ = 4.950, *P* = 0.031, *η*^2^ = 0.092).

There were marginally significant interactions among benzodiazepines, stimulus, and task during the early P3 phase (450–550 ms) for the central area (*F*_2, 49_ = 3.156, *P* = 0.051, *η*^2^ = 0.114) and significant interactions for occipital area (*F*_2, 49_ = 3.347, *P* = 0.043, *η*^2^ = 0.120). For the later P3 phase (550–650 ms) there were marginally significant interactions for the central area (*F*_2, 49_ = 2.540, *P* = 0.089, *η*^2^ = 0.094) and occipital area (*F*_2, 49_ = 2.669, *P* = 0.079, *η*^2^ = 0.098). Pairwise comparison showed that P3 amplitudes at 450–550 ms induced by pain depictions were more positive than responses to neutral images in HCs for the central area (*F*_1, 49_ = 12.796, *P* = 0.001, *η*^2^ = 0.207) and occipital area (*F*_1, 49_ = 9.908, *P* = 0.003, *η*^2^ = 0.168). Positive amplitudes for P3 (550–650 ms) were higher in HCs for the central area (*F*_1, 49_ = 19.471, *P* < 0.001, *η*^2^ = 0.284) and occipital area (*F*_1, 49_ = 13.294, *P* = 0.001, *η*^2^ = 0.213). Finally, P3 (450–550 ms) amplitudes were higher with marginal significance in patients not receiving benzodiazepines for the central area (*F*_1, 49_ = 3.026, *P* = 0.088, *η*^2^ = 0.058) and occipital area (*F*_1, 49_ = 3.460, *P* = 0.069, *η*^2^ = 0.066), and P3 (550–650 ms) amplitudes were higher in these patients for the central area (*F*_1, 49_ = 4.135, *P* = 0.047, *η*^2^ = 0.078) and occipital area (*F*_1, 49_ = 4.836, *P* = 0.033, *η*^2^ = 0.090). Patients currently receiving benzodiazepines showed no differences in P3 amplitudes (*P* > 0.5).

### Correlation analysis

Pearson correlation analysis showed significant negative correlations between scores on the Young Mania Rating Scale (YMRS) and P3 (550–650 ms) difference waves in the pain task for the frontal area (*r*_30_ = −0.497, *P* = 0.005) and central area (*r*_30_ = −0.377, *P* = 0.040), and a marginally significant correlation for the occipital area (*r*_30_ = −0.347, *P* = 0.060)]. Scores on the Hamilton Depression Scale (HAMD) did not show any significant correlation with either early or late difference waves, but the scores were positively correlated with personal distress (*r* (30) = 0.544, *P* = 0.002).

## Discussion

The main aim of this study was to compare neural processing of pain empathy between BD patients and HCs using ERP analysis. Bipolar disease patients exhibited abnormal N1 and lower P3 amplitudes evoked by depictions of pain in others compared to neutral images during the pain judgment task. Source localization analysis showed higher current source density from the ACC of HCs when presented with pain depictions compared to neutral images during the pain judgment task. However, this difference was not observed in BD patients. Self-reported empathic concern and perspective taking scores were also lower in BD patients. The present study extends the findings of previous behavioural studies by providing solid evidence of impaired empathy in BD at the neural processing level. In addition, manic symptom scores were negatively correlated with P3 difference waves (pain depiction – neutral image condition), strongly suggesting that the aberrant neural processing of pain empathy in BD is dependent on current disease state.

We detected an N1 component of the ERP response corresponding to early processing of pain empathy, with a trend showing more positive-amplitude N1 waves in response to depictions of other’s pain compared to neutral images in HCs, consistent with previous studies^23^,^24^,^33^. In contrast, N1 amplitudes observed in BD patients were less positive in response to pain depiction than neutral images during the pain judgment task. The early ERP component N1 may be a discriminative index for bottom-up sensory mechanisms sensitive to stimulus salience^34^. The difference in N1 amplitude between pain depiction and neutral image conditions in the pain empathy task is believed to reflect differences in automatic visual processing relating to emotionally evocative stimuli compared to neutral stimuli^23^,^24^. Our results suggest that BD patients have deficit in early processing of pain empathy, indicating lower emotional arousal at the sight of pain in others compared to healthy controls. Indeed, BD patients were previously found to present with an abnormality in early visual processing^29^. However, the results of N1 analysis may be confounded by medications as discussed below.

We also detected later ERP components P3 (450–550 ms) and P3 (550–650 ms) that differed in amplitude between groups. Consistent with previous studies, the positive P3 signal was larger in response to pain depiction than neutral images during the pain judgment task^23^,^24^,^33^. The P3 component is widely considered to reflect attentional processes, stimulus perception, evaluation, and classification at the conscious level^35^, independent of response selection and execution^36^. Stimuli that are more arousing, salient, and motivationally significant usually elicit larger P3 responses. Stimuli associated with pain convey information important for survival and thus demand greater allocation of attention^37^. However, P3 amplitudes during pain judgment were smaller in BD patients than HCs, while the P3 responses to neutral images were similar between the two groups. Thus, the differential response (discrimination of pain depictions vs. neutral images) was smaller in BD patients at the later stage neural processing. The smaller differential response suggests that BD patients do not allocate sufficient attentional resources for judging other’s pain compared to HCs and thus have difficulty evaluating and empathizing with other’s pain. This weaker neural activation is consistent with previous results, indicating that BD patients were less sensitive to other people’s pain on the neurophysiological level. BD patients often exhibit deficits in emotion identification and perspective taking^8^,^9^,^10^,^11^. Thus, they may have difficulty empathizing with other people’s pain because of their inability to accurately identify relevant cues and to take the perspective of others^11^,^18^,^19^.

Bipolar disease patients also exhibit P3 deficits in other cognitive tasks using a variety of target stimuli^31^. As painful conditions occur infrequently in everyday life and demand more attention due to their greater relevance to survival, painful stimuli may be regarded as predominant target stimuli. Therefore, P3 deficits on pain empathy tasks may suggest associations between pain empathy and other cognitive domains in BD patients. Further studies comparing different forms of P3-associated processing in BD patients may reveal these additional deficits. The P3 response has been compared between BD and schizophrenia patients^38^. While BD patients generally show less empathy impairment than schizophrenia patients^20^, both groups exhibited decreased P3 responses to others’ pain in pain empathy tasks, suggesting some shared neuropathological mechanisms^28^. Further empathy-related ERP studies including both BD and schizophrenia groups may illuminate shared as well as disease-specific mechanisms for social deficits.

We also found significant region-specific negative correlations between P3 difference waves and manic symptoms. That is, patients with manic symptoms had lower empathy for other people’s pain compared to non-manic patients due to specific processing deficits. This is consistent with previous findings that patients with more severe manic symptoms or patients in manic states were less able to identify the negative emotions of others^39^,^40^. One study reported that manic patients showed a selective negative facial emotion processing deficit associated with decreased P3 amplitude^41^. The severity of manic symptoms may reduce the normal attentional bias to distress, including pain of others. Correlational analysis strongly suggests that manic symptoms contribute to the neural processing deficits (manifested by decreased P3 amplitudes) in the pain depiction condition. In contrast, there was no significant correlation between HAMD score and P3 difference wave amplitude, suggesting that depressive symptoms do not influence the deficit of pain empathy in BD patients, as also indicated by self-report results. On the other hand, HAMD scores were positively correlated with self-reported personal distress, as reported previously^18^. Depressed patients may experience more self-oriented empathy when faced with other’s distress as they tend to focus on the self rather than others^42^. However, the present results are preliminary as the sample size was small and we did not compare differences among BD patient subgroups. Future studies are required to confirm the association between low pain empathy and symptom severity.

Using sLORETA source localization analysis during a late ERP time window, we found that BD patients exhibited little difference in ACC current source density between pain and neutral judgment conditions. Previous research demonstrated that the ACC plays an important role in the processing of pain empathy^13^,^21^. Our source localization analysis thus confirms that BD patients show abnormal activation in the ACC during the processing of pain empathy. While source localization was based on a mathematical model rather than direct physiological measures, our results provide direction for further studies using higher spatial resolution methods to investigate differences in neural activation patterns among BD patient subtypes and HCs during tasks that should evoke empathy. Furthermore, as previous treatment attempts have targeted specific neuroanatomic structures, such as transcranial magnetic stimulation^43^,^44^, these findings may help define the most therapeutically effective targets for activity modulation.

Medications also influence ERP amplitudes^45^. For instance, benzodiazepines consistently reduce ERP amplitudes^46^. Clonazepam reduced the auditory evoked potential components N1 and P3 compared to placebo^47^. In the present study, we also found that benzodiazepines (usually clonazepam only) had a negative impact on N1 and P3 components in the pain task. Less positive N1 amplitude in the pain condition strongly suggests that benzodiazepines diminish conditional discrimination reflected by N1, likely by suppressing emotional arousal. This may be an important confounding influence on the difference in N1 between BD patients and HCs. Furthermore, benzodiazepines decreased the P3 difference waves during 450–550 ms and 550–650 ms periods. However, it is currently difficult to conclude that group differences in late P3 are due to medication. Patients not currently taking benzodiazepines also showed decreased difference waves compared to HCs. Previous study found no effect of antipsychotics on N1 amplitude in schizophrenia patients^48^, but few studies have investigated such effects in BD patients. In the present study, antipsychotics were found to have a beneficial effect on pain empathy in BD patients, as patients not taking antipsychotics showed smaller N1 difference waves (i.e., poorer discrimination) compared to both patients currently on antipsychotics and HCs. Antidepressants and mood stabilizers also suppressed N1 amplitude. However, there are few studies assessing the effects of these two medications alone or in combination on early ERP components in BD patients. The observed effects may have resulted from a combination of different medications. Thus, studies of patients on single medications are required to assess the underlying mechanism.

The present investigation had some limitations. First, our sample size was relatively small, increasing the likelihood of type II errors. Second, the sample included patients with varying courses of illness so we could not distinguish whether the neural processing abnormalities in BD patients are state- or trait-dependent. Further studies involving larger numbers of subjects in varying states of illness are required to confirm these preliminary findings and clarify the relationship between symptom severity and the neural mechanisms of pain empathy. Third, all patients participating in the present study were currently taking medications, which proved to be an important confound on the ERP results. Although we made efforts to analyse the effects of each medication, some patients were taking combinations. Future studies should therefore be conducted with stricter control of concomitant medication. Finally, the spatial resolution of the ERP technique was comparatively low, providing only limited evidence for differences in neural activation pattern between patients and controls. However, the main goal of this study was to investigate differences in the temporal dynamics of empathic processing between BD patients and HCs, which was achieved. Further studies using high-resolution neuroimaging techniques such as functional MRI are needed to reveal the neural activation patterns associated with pain empathy in BD.

In conclusion, our study provides preliminary but compelling evidence for a pain empathy deficit in BD patients at the neurophysiological level using ERP recordings during a well validated empathy paradigm. The results showed that late (conscious) and potentially also early (automatic) neural processing stages of pain empathy were impaired and that ACC activity while judging pain in others was reduced in BD patients. In addition, self-reported empathy scores were lower in BD patients than HCs. Taken together, the present results demonstrate impaired empathy at the neurophysiological level in patients with BD. YMRS scores were negatively correlated with difference waves, providing the first indication that altered pain empathy in BD may be state-dependent. This deficit in empathy may explain the poor interpersonal interactions observed in many BD patients and suggests that BD patients may benefit from psychotherapy targeting empathy.

## Methods

### Subjects

Twenty-two outpatients and eight inpatients diagnosed with BD using the 10th version of the International Classification of Diseases (ICD-10) were recruited from the Anhui Mental Health Centre. Twenty-three HCs were recruited by advertisements from the community and matched to the patients for age, sex ratio, and education. The HCs had no personal or family history of psychosis. Exclusion criteria for patients and HCs were: i) history of electroconvulsive therapy in the previous six months, ii) substance abuse based on ICD-10 criteria in the previous six months, iii) current or history of nervous system disease, iv) history of brain injury.

Patients were currently treated with medications. All patients were taking mood stabilizers (n = 21), antipsychotics (n = 18), and/or antidepressants (n = 19), either individually or in combinations of two or three of these medications. Some patients were also taking benzodiazepines (n = 11) in addition to these other drugs. Patients were in different disease states, including depressed, manic, and remitted. We used the 17-item HAMD^49^ and YMRS^50^ to assess depressive and manic symptoms (HAMD, 10.67 ± 8.231; YMRS, 5.00 ± 6.963), respectively.

All subjects signed an informed consent form for the study. All study procedures were approved by the Anhui Medical University Ethics Committee and conducted according to the Helsinki Declaration (1975 and subsequent revisions).

### Stimuli

**Figure 4:**
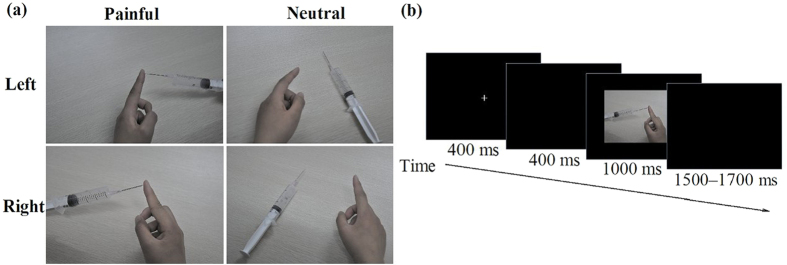
Illustration of the stimuli and experimental procedure used in the current study. (**a**) Stimuli used in this study; (**b**) Timing of events in one trial.

As previously reported^51^, 140 digital pictures were presented. Pictures involved a body part in painful or neutral conditions without faces shown, and were taken from the first-person perspective. Pictures were divided into 35 situations and each situation contained four conditions: i) left body part in painful situation, ii) left body part in neutral situation, iii) right body part in painful situation, and iv) right body part in neutral situation. The pictures in painful conditions depicted incidents that may happen in everyday life (such as a hand trapped in a door). Pictures in the four conditions had identical physical properties (i.e., context, brightness, and contrast) (Fig. 4). Pain intensity of all pictures was rated by an independent group of 33 subjects on a 5-point scale from 1 (“not painful at all”) to 5 (“extremely painful”).

### Procedure

Before electroencephalograph (EEG) recordings, subjects were assessed empathy with the Chinese IRI^52^,^53^ and neurocognition with Verbal Fluency, Digit Span Forward, Digit Span Backward, Stroop and the Montreal Cognitive Assessment^54^,^55^.

A block design task consisting of two blocks was administered in the pain empathy task, as described in previous studies^23^,^51^. In the pain task block, subjects were asked to judge whether the person in the picture felt pain. In the laterality task block, subjects were asked to judge the laterality of the body part. Subjects were asked to respond as quickly and accurately as possible while recording EEG. The order of the two blocks was counterbalanced in each group of subjects. Each block consisted of 120 trials (60 painful and 60 matched neutral pictures). Each trial was initiated with a fixation cross, followed by a blank screen for 400 ms. Next, a picture was presented for 1000 ms, followed by a blank screen allowing subjects to respond within a random period between 1500 and 1700 ms. All pictures were randomly presented at the centre of a black background on a computer screen. The same pictures were used in both blocks. Before each block, subjects underwent a practice session of 20 trials using different pictures from those presented in the experiment. Subjects were asked to rate the level of pain felt by the person in all pictures immediately after the EEG recording using a 5-point scale from 1 (“not painful at all”) to 5 (“extremely painful”).

### EEG data recording and analysis

EEG data were recorded from 64 tin electrodes placed on the scalp according to the extended International 10/20 system using a Neuroscan recording system (Neuro Scan, Sterling, VA, USA). Four electrodes were used to measure electrooculogram (EOG), with vertical EOG recorded above and below the left eye and horizontal EOG on the outer canthus of the left and right eye. EEG signals were recorded using a left mastoid electrode as the online reference. All electrode impedances were maintained below 10 kΩ. EEG and EOG activities were amplified with 0.01–100 Hz band-pass filtering and continuously sampled at 500 Hz/channel.

Offline processing was implemented in EEGLAB^56^ implemented in Matlab 2015a (Mathworks, Natick, MA, USA). The data were re-referenced to the average of left and right mastoids and down-sampled at 250 Hz. A high-pass 0.02 Hz filter was used to remove slow drift. Bad and artefactual electrodes were detected by an algorithm developed by Nima Bigdely-Shamlo (retrieved from https://github.com/bigdelys/pre_ICA_cleaning). Continuous data of every channel were cut into 1-s segments. Segments were considered artefactual when the correlation coefficient with others channels was lower than 0.4. If the proportion of artefactual segments from a channel was higher than 1%, all data from that channel were rejected. The preprocessed data were segmented into 3000 ms epochs covering 1000 ms before to 2000 ms after onset of image presentation.

Artefactual epochs were identified and removed based on a) abnormal spectral characteristics of high frequency noise (rejspec; 20–40; <−35 or >35 dB), b) abnormal trends (rejtrend; slope > 200 μV with R2 > 0.2), c) abnormal amplitude (threshold −300 μV or +300 μV), d) improbable data using joint probability (jointprob, 8 standard deviation (SD) for single channel and 4 SD for all channels), or e) abnormal distributions (rejkurt; 8 SD for single channel and 4 SD for all channels). Data from electrodes responsible for more than 10% of rejected epochs were rejected. Then, independent component analysis (infomax) was conducted on 1 Hz high-pass filtered data and the resulting weight matrices were applied to the 0.02 Hz high-passed data. Independent components corresponding to EOG, electromyogram and electrocardiogram were removed. On average, 58 (SD 3) channels and 55 (SD 4) independent components were retained per subject. The mean fraction of excluded trials was 4.11% (SD 2.1%) in the patient group and 5.06% (SD 3.5%) in HCs. Rejection rate did not differ significantly between groups (*t*_47_ = 0.65, *P* = 0.52).

The ERP waveforms were time-locked to stimulus onset and epoched to 200 ms pre-stimulus and 1000 ms post-stimulus. Only the N1 and P3 components were analysed in this experiment. N1 was defined as the mean amplitude of the 100 ms time window centred around peak N1 ground average waveform. P3 was calculated as the mean amplitude of two 100 ms time windows (450–550 ms and 550–650 ms) centred around peak P3 ground average waveforms. Statistical analyses were conducted on frontal (AF3/AF4, Fz, FCz, F3/F4, F5/F6, F7/F8, FC1/FC2, FC3/FC4, FC5/FC6), central (Cz, C1/C2, C3/C4 and C5/C6), and parietal (Pz and P3/P4, CP5/CP6, CP3/CP4, CP1/CP2, CPZ, P7/P8) electrode sites.

### Source-localization analysis

Standard low-resolution electromagnetic tomography (sLORETA, v20151222) analysis was applied to calculate the cerebral generators of pain empathy processing based on a subject-specific boundary element model applied to the Montreal Neurological Institute 152 template^57^,^58^,^59^. We estimated current density distributions in the cortical grey matter and hippocampus from the digitised Montreal Neurological Institute atlas with 6, 239 voxels at 5 mm spatial resolution. For each voxel, sLORETA values represent the power of the squared magnitude of the computed intracerebral current density and were then log-transformed within subjects before statistical analysis.

To detect the neural generators of pain empathy processing in the HCs and BD groups, voxel-based whole brain sLORETA images were compared between pain and neutral image conditions. The sLORETA built-in voxel-wise randomisation tests (5000 permutations) based on statistical non-parametric mapping methodology were used to yield a whole-brain statistical correction^60^.

### Statistical analysis

All data were analysed using IBM SPSS 16.0 (IBM Corp., Armonk, NY, USA). One sample K-S tests were conducted on all continuous data. Independent samples *t*-test were used to assess group differences in age, years of education, neurocognitive test scores, pain ratings and Chinese IRI subscale scores. Chi-square tests were used to assess the difference in sex ratio between groups. Repeated measures analysis of variance tests were conducted on RTs, accuracy of task response (pain task and laterality task) and stimulus (pain and neutral) as within-subject factors, and group (BD patients and HCs) as a between-subject factor. RM-ANCOVA tests were conducted on the average amplitude of each ERP component measured from frontal, central, and occipital areas, with task and stimulus as within-subject factors, group as a between-subject factor, and pain ratings as a covariate.

A potential confounding factor is the different types of medications currently used by the patients. Medications were coded as ‘on’ or ‘off’ for each patient. RM-ANCOVA tests were conducted with task, stimulus as within-subject factors, group (patients on medication, patients off medication, and HCs) as a between-subjects factor and pain ratings as a covariate.

Greenhouse-Geisser correction was used to correct *P* values, and the Bonferroni method was used for multiple comparisons. Bivariate Pearson correlations were calculated to examine the association strengths among difference waves, Chinese IRI and symptom severity as measured by YMRS and HAMD scores. A two-tailed *P* < 0.05 was considered significant.

## Additional Information

**How to cite this article**: Yang, J. *et al*. Decreased empathy response to other people’s pain in bipolar disorder: evidence from an event-related potential study. *Sci. Rep.* **7**, 39903; doi: 10.1038/srep39903 (2017).

**Publisher's note:** Springer Nature remains neutral with regard to jurisdictional claims in published maps and institutional affiliations.

## Figures and Tables

**Table 1:** Demographic data, neurocognitive tests and self-reported empathy between groups.

	Bipolar disorder		Healthy control		*t*/*χ*^2^	*P*	*P* of K-S Tests (BD/HCs)
	M/female	SD	M/female	SD			
Demographic data							
Age(years)	30.97	8.65	33.45	7.19	−1.061	0.294	0.653/0.522
Sex ratio	19	N/A	17	N/A	0.669	0.413	N/A
Education(years)	12.97	3.51	12.68	2.64	0.320	0.751	0.113/0.399
Neurocognitive tests							
Verbal Fluency	19.22	3.67	20.29	4.68	−0.935	0.354	0.814/0.515
Digit Span Forward	8.11	1.09	8.54	0.78	−1.598	0.116	0.181/0.294
Digit Span Backward	5.41	1.52	6.05	0.86	−2.000	0.051	0.333/0.088
Stroop	14.59	9.23	10.68	5.11	1.826	0.074	0.648/0.123
MoCA	25.96	2.93	27.85	1.10	−3.244	0.002	0.076/0.069
Pain ratings	1.94	0.67	2.20	0.45	−1.683	0.099	0.919/0.944
IRI-C							
Perspective Taking	9.17	5.38	12.19	3.79	−2.29	0.026	0.528/0.915
Fantasy	12.45	4.78	15.19	5.68	−1.904	0.063	0.945/0.622
Empathic Concern	14.97	5.19	17.94	3.36	−2.389	0.021	0.980/0.889
Personal Distress	9.14	5.64	8.43	5.11	0.471	0.640	0.854/0.668

M = mean; SD = standard deviations. BD = bipolar disorder; HCs = healthy controls; Neurocognitive tests: MoCA = Montreal Cognitive Assessment. Pain ratings were referred to discrimination ratings of pain-depicting and neutral images. Empathy measures: IRI-C = Chinese version of Interpersonal Reactivity Index.

**Table 2:** Reaction time for correct response and accuracy of bipolar disorder patients and healthy controls.

	Bipolar disorder				Healthy control			
	Neutral stimuli		Pain stimuli		Neutral stimuli		Pain stimuli	
	M	SD	M	SD	M	SD	M	SD
Pain task								
** **Accuracy	0.89	0.06	0.76	0.21	0.89	0.06	0.81	0.11
** **RTs (ms)	876.50	170.01	896.65	147.22	748.48	89.05	757.44	90.89
Laterality task								
** **Accuracy	0.95	0.05	0.93	0.05	0.97	0.03	0.93	0.03
** **RTs (ms)	723.93	110.14	771.63	118.30	637.98	71.10	670.10	60.35

M = mean; SD = standard deviations. RTs: reaction time for correct response.
